# Supplementary material for: Global translation during early development depends on the essential transcription factor PRDM10
Source: Nat Commun. 2020 Jul 17;11:3603. doi: 10.1038/s41467-020-17304-3 (PMC7368010; doi:10.1038/s41467-020-17304-3)
Supplement: Supplementary file 1 — Supplementary Information [file 41467_2020_17304_MOESM1_ESM.pdf]

Han *et al.*, Supplementary Information

Global translation during early development depends on the essential transcription factor PRDM10

Supplementary Figure 1

a

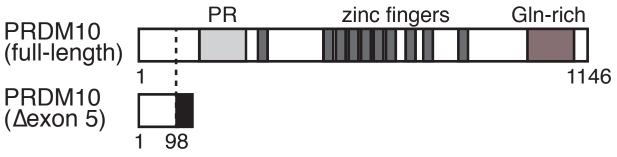

b

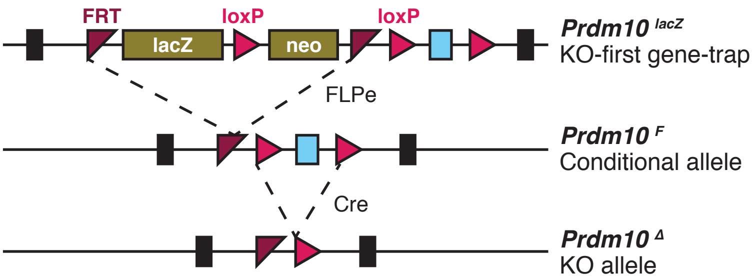

c

Genotyping of embryos from *Prdm10*$^{Δ/+}$ X *Prdm10*$^{Δ/+}$ crosses

Stage	+/+	Δ/+	Δ/Δ	n.d.	total (n)	P-value	
E3.5	34 (30.1%)	51 (45.1%)	28 (24.8%)	N/A	113	0.6332	n.s
E4.5	25 (29.4%)	46 (54.1%)	14 (16.5%)	N/A	85	0.3967	n.s
E7.5	23 (28.8%)	37 (46.3%)	0 (0%)	20 (25.0%)	80	0.0002	***
E12.5	10 (33.3%)	13 (43.3%)	0 (0%)	7 (23.3%)	30	0.0299	*

n.d., only empty deciduomas detected, embryo genotype not determined.

d

Morphological scoring of E3.5 embryos

Category	+/+	Δ/+	Δ/Δ
morula	8 (29.6%)	6 (15.4%)	18 (85.7%)
partially expanded blastocyst	9 (33.3%)	16 (41.0%)	2 (9.5%)
fully expanded blastocyst	10 (37.0%)	17 (43.6%)	1 (4.8%)
total (n):	27 (100%)	39 (100%)	21 (100%)

e

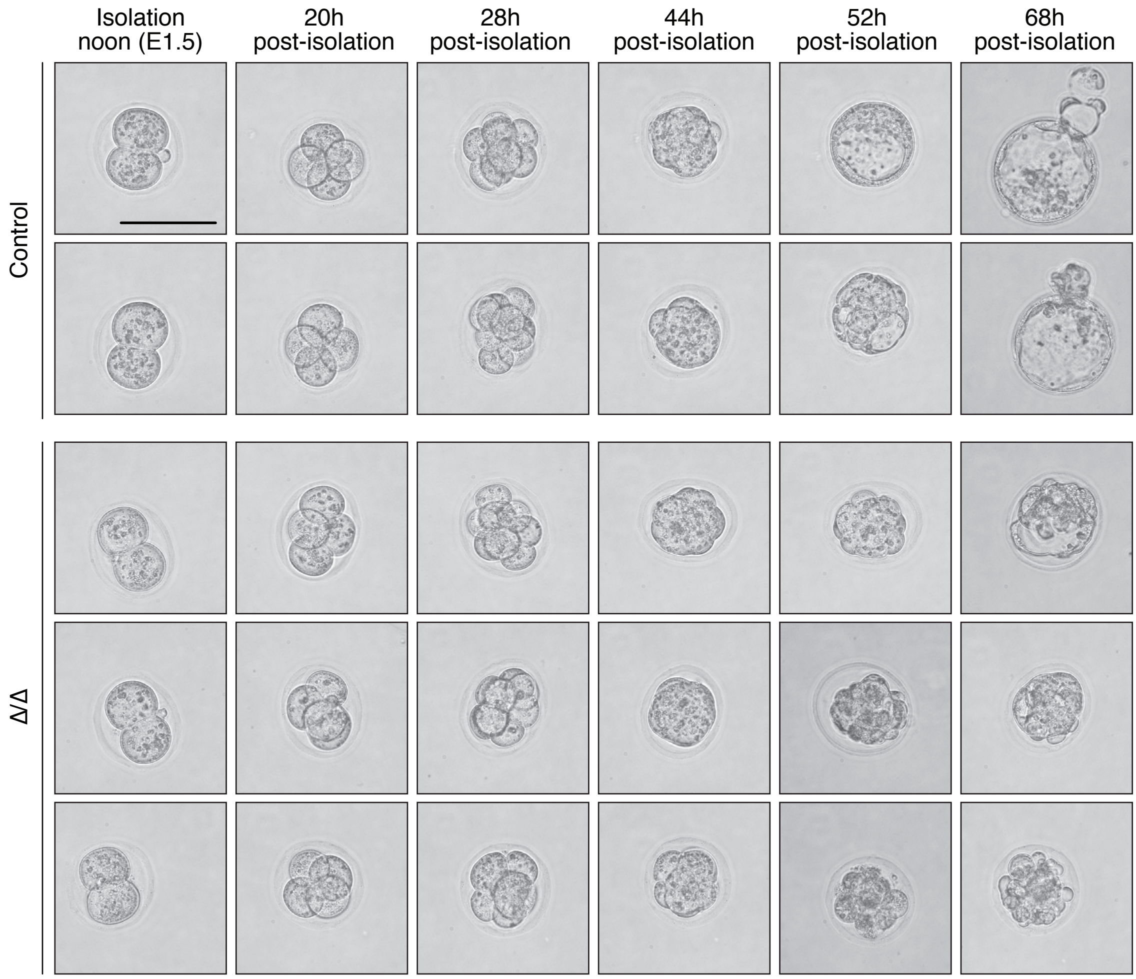

Supplementary Figure 1. PRDM10 deletion *in vivo* results in pre-implantation embryonic lethality.

(a) Schematic depicting the N-terminal PR domain, C2H2 zinc fingers and C-terminal Gln-rich domain of PRDM10 (*top*); prematurely truncated protein generated by out-of-frame deletion of exon 5 (*bottom*). Numbers indicate amino acid position.

(b) Mouse model for *Prdm10* deletion. A *Prdm10* 'knockout-first' gene-trap allele (*Prdm10*^{lacZ}) was converted to a conditional (*Prdm10*^{F}) allele by FLPe-mediated recombination, which was then used to generate a null (*Prdm10*$^{\Delta}$) allele by Cre-mediated recombination.

(c) Summary of embryo numbers for each genotype from *Prdm10*$^{\Delta/+}$ intercrosses. Resorptions detected at E7.5 and E12.5 are indicated as n.d. Chi-square test for significant deviation from expected Mendelian distribution; *$P < 0.05$, ***$P < 0.001$.

(d) Summary of embryo numbers for each genotype from *Prdm10*$^{\Delta/+}$ intercrosses, isolated at E3.5 and scored by morphology into 3 phenotypic categories.

(e) Representative images of control (*Prdm10*$^{+/+}$*, Prdm10*$^{\Delta/+}$) and mutant (*Prdm10*$^{\Delta/\Delta}$) embryos isolated at E1.5 (2-cell stage) and cultured *ex vivo*. n = 36 (control), n = 14 (*Prdm10*$^{\Delta/\Delta}$). Scale bar: 100 μm.

Supplementary Figure 2

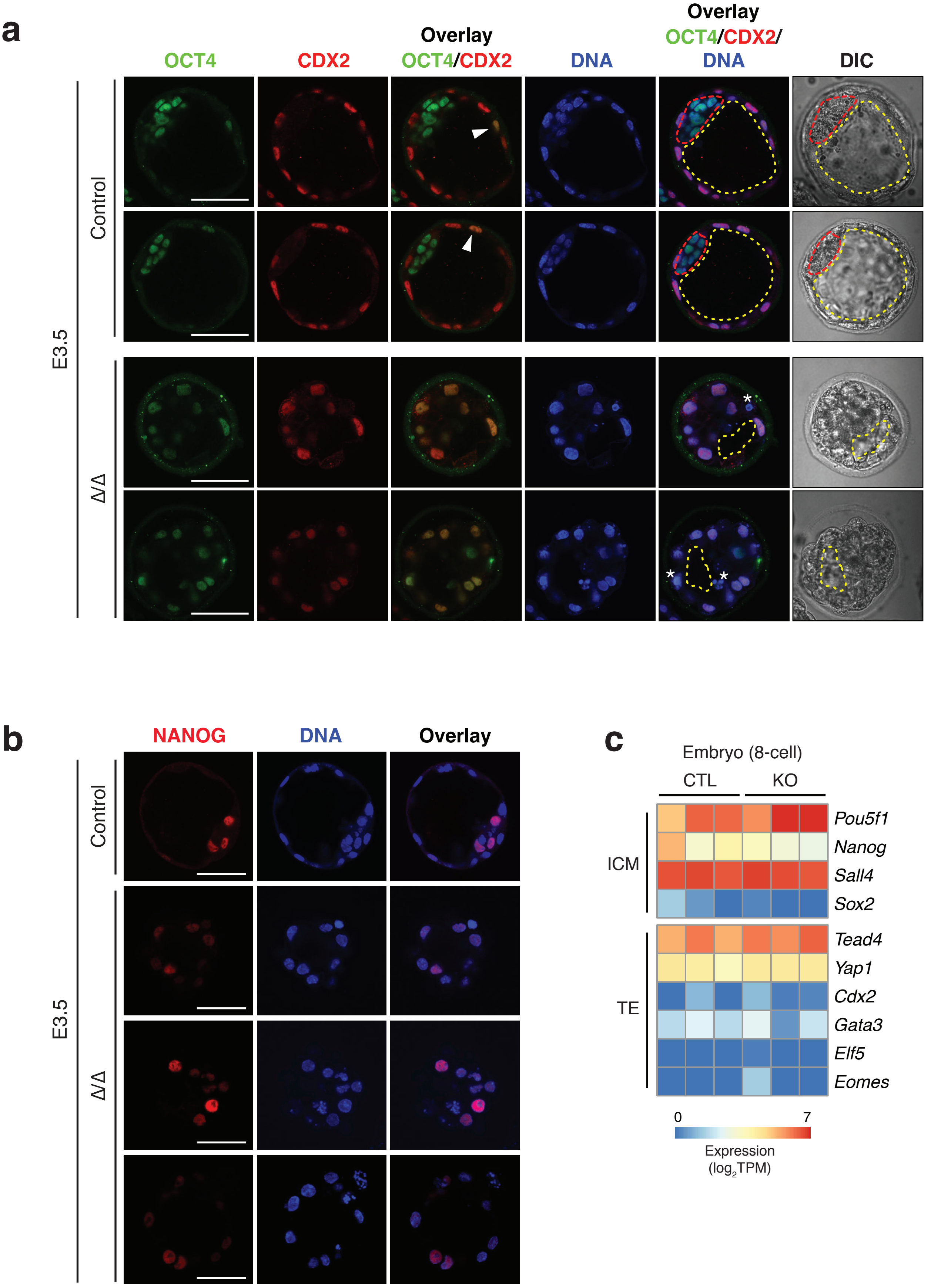

Supplementary Figure 2. Expression of ICM and TE lineage markers in *Prdm10*$^{\Delta/\Delta}$ embryos.

(a) Representative confocal images of control (*Prdm10*$^{+/+}$, *Prdm10*$^{\Delta/+}$) and mutant (*Prdm10*$^{\Delta/\Delta}$) E3.5 embryos stained for OCT4 and CDX2. Nuclei are visualized by DAPI staining. Blastocoel (yellow dashed line) and inner cell mass (red dashed line) are outlined. White arrowheads identify cells co-expressing OCT4 and CDX2. Asterisks label pyknotic, fragmented nuclei indicative of apoptotic cells. *n* = 24 (control), *n* = 10 (*Prdm10*$^{\Delta/\Delta}$). Scale bar: 50 μm.

(b) Representative confocal images of control and mutant E3.5 embryos stained for NANOG. Nuclei are visualized by DAPI staining. Scale bar: 50 μm.

(c) Heatmap depicting expression of selected inner cell mass (ICM) and trophectoderm (TE) markers in control (CTL) vs. *Prdm10*$^{\Delta/\Delta}$ (KO) 8-cell stage embryos. Expression values shown as $\log_2$(TPM + 1).

Supplementary Figure 3

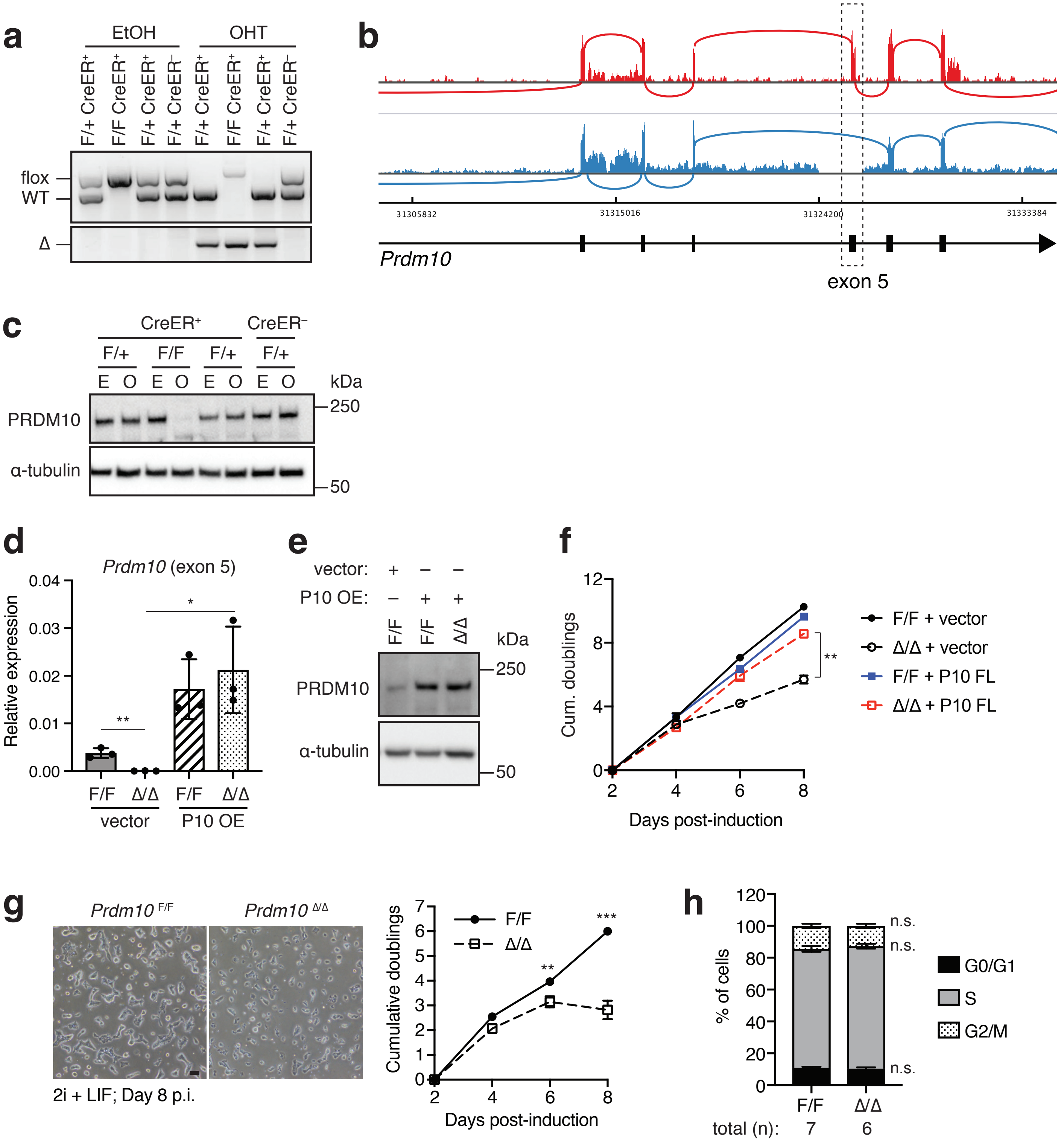

Supplementary Figure 3. Molecular validation and phenotypic characterization of PRDM10 deletion in mESCs.

(a) PCR analysis of genomic DNA from *Prdm10*$^{F/F}$; CreER^{T2} and *Prdm10*$^{F/+}$; CreER^{T2} mESCs show efficient Cre-mediated recombination after 24 h induction with 50 nM 4-OHT. No recombination is observed in vehicle (EtOH)-treated cells or CreER^{T2}-negative cells.

(b) Sashimi plot of aligned RNA-seq reads from *Prdm10*$^{F/F}$ and *Prdm10*$^{\Delta/\Delta}$ mESCs at Day 2 post-deletion. The complete excision of exon 5 is accompanied by aberrant splicing of exon 4 to 6, resulting in a frameshifted *Prdm10*$^{\Delta exon5}$ transcript.

(c) Western blot analysis of PRDM10 protein levels in wild-type (F/+ CreER$^-$), heterozygous (F/+ CreER$^+$) and homozygous mutant (F/F CreER$^+$) mESCs. Cells were collected at 3 days post-treatment with EtOH (E) or 4-OHT (O). Loading control: α-tubulin.

(d) qRT-PCR analysis of *Prdm10* exon 5 expression in *Prdm10*$^{F/F}$; CreER^{T2} mESCs overexpressing full-length Prdm10 (P10 OE) or empty vector, at 4 days after EtOH treatment (F/F) or OHT-induced recombination (Δ/Δ). Expression shown normalized to *Ubb.*

(e) Western blot showing PRDM10 protein expression in *Prdm10*$^{F/F}$ and *Prdm10*$^{\Delta/\Delta}$ cells stably transfected with empty vector (EV) or *Prdm10* (P10 OE). Loading control, α-tubulin.

(f) Rescue of growth defect in *Prdm10*$^{\Delta/\Delta}$ mESCs by exogenous PRDM10 expression (red dashed line) compared to vector control (black dashed line). Y-axis:

cumulative population doublings; *n* = 3 samples, representative data shown from one out of three independent experiments.

(g) Growth analysis of PRDM10-deficient mESCs cultured in serum-free 2i/LIF conditions. Cells were counted and passaged at constant density every two days up to Day 8 post-induction. Representative brightfield images of $Prdm10^{F/F}$ and $Prdm10^{\Delta/\Delta}$ mESC colonies at Day 8 (*left*); cumulative population doublings over time (*right*); *n* = 3 samples, representative data shown from one of two independent experiments.

(h) Cell cycle distribution of $Prdm10^{F/F}$ or $Prdm10^{\Delta/\Delta}$ mESCs, assessed by flow cytometry at Day 5 post-induction. Data from 6–7 biological replicates pooled across three independent experiments.

Data are presented as mean ± s.d. (**d, f, g** and **h**). **P* < 0.05, ***P* < 0.01, ****P* < 0.001; two-tailed unpaired Student's *t*-test (**d**, **g** and **h**), two-way ANOVA with Tukey's multiple comparisons test (**f**).

Supplementary Figure 4

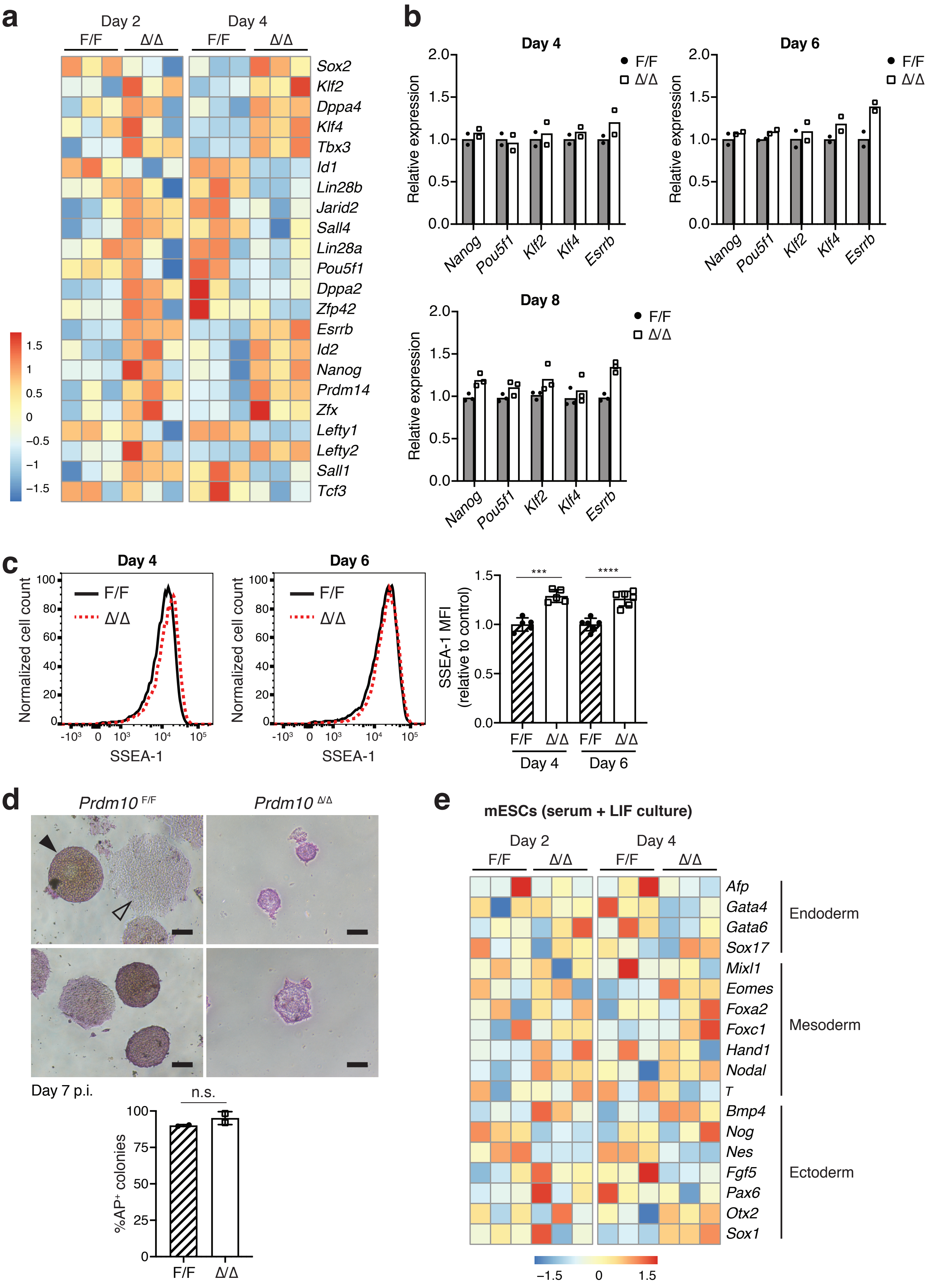

Supplementary Figure 4. PRDM10 is dispensable for mESC pluripotency.

(a) Heatmap visualization of genes associated with stem cell pluripotency in *Prdm10*$^{F/F}$ and *Prdm10*$^{\Delta/\Delta}$ mESCs, as measured by RNA-seq at Day 2 and Day 4 post-induction. Scale: row-normalized Z-score.

(b) qRT-PCR analysis of *Prdm10*$^{\Delta/\Delta}$ mESCs compared to *Prdm10*$^{F/F}$ controls at Day 4 to Day 8 post-deletion reveals no significant decrease in expression of ESC pluripotency markers *Nanog*, *Pou5f1* (Oct4), *Klf2*, *Klf4* and *Esrrb*. Expression data normalized to *Ubb* and reported relative to control cells. Data shown as mean ± s.d. of 2–3 biological replicates, each the mean of two technical qPCR replicates.

(c) Flow cytometric analysis of SSEA-1 surface expression on *Prdm10*$^{\Delta/\Delta}$ mESCs compared to *Prdm10*$^{F/F}$ controls at Day 4 (n = 5) and Day 6 (n = 6) post-deletion. MFI: geometric mean fluorescence intensity, normalized to controls within each experiment. Data combined from two independent experiments and presented as mean ± s.d. ***$P < 0.001$, ****$P < 0.0001$, two-tailed unpaired Student's *t*-test.

(d) Representative images of *Prdm10*$^{F/F}$ and *Prdm10*$^{\Delta/\Delta}$ mESC colonies stained for alkaline phosphatase (AP) activity at Day 7 post-induction, with examples of AP^{+} (solid arrowhead) and AP^{-} (open arrowhead) colonies highlighted (*top*). Percentage of AP^{+} colonies in *Prdm10*$^{F/F}$ vs. *Prdm10*$^{\Delta/\Delta}$ cultures quantified across two independent experiments (*bottom*). n.s., not significant, two-tailed unpaired Student's *t*-test. Scale bar: 100 µm.

(e) Heatmap depicting expression of selected differentiation markers for endoderm, mesoderm and ectoderm lineages in *Prdm10*$^{F/F}$ and *Prdm10*$^{\Delta/\Delta}$ mESCs, as

measured by RNA-seq at Day 2 and Day 4 post-induction. Scale: row-normalized Z-score.

Supplementary Figure 5

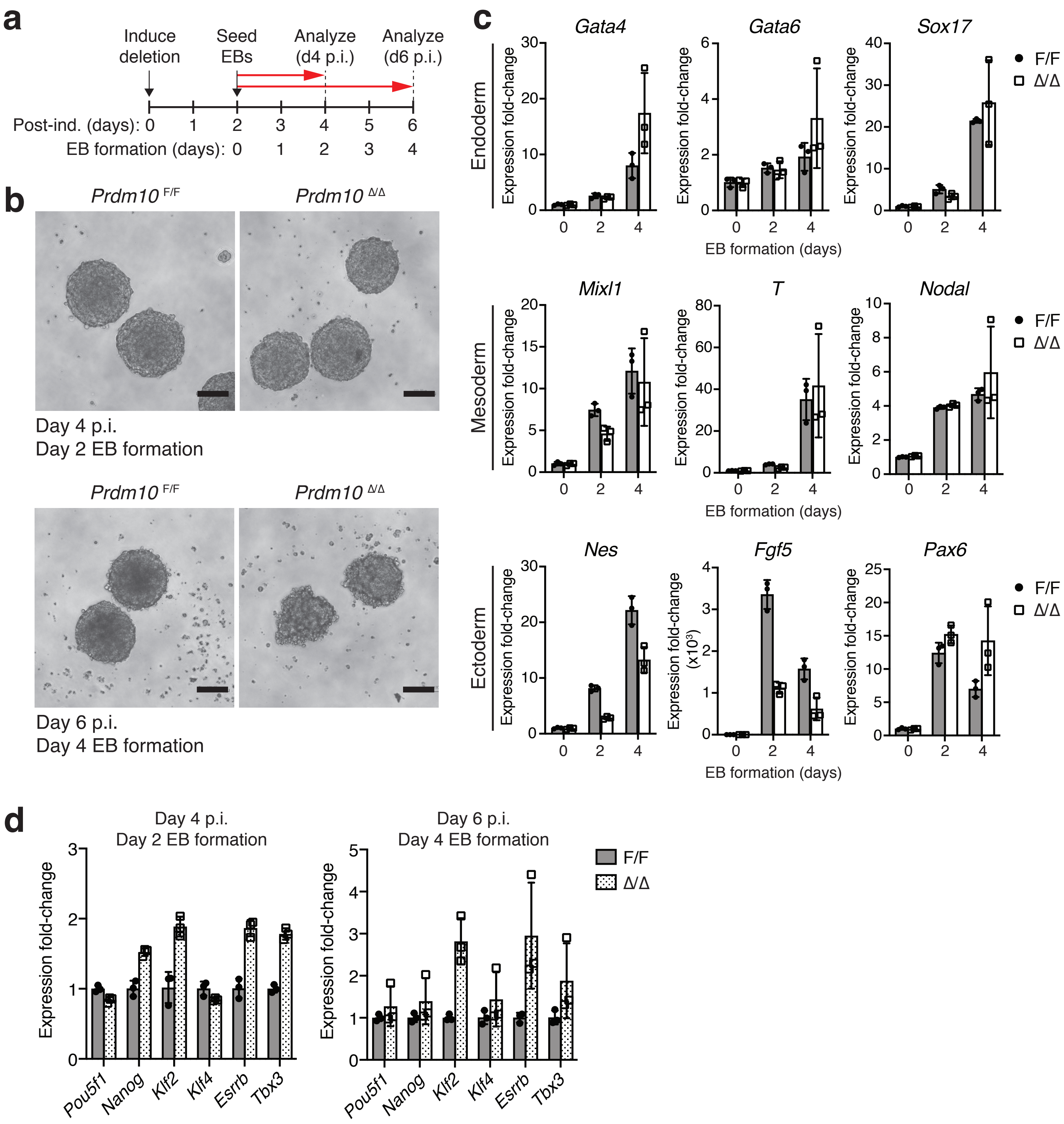

Supplementary Figure 5. PRDM10 is not required for mESC differentiation.

(a) Experimental set-up for embryoid body (EB) differentiation and analysis. Undifferentiated mESCs were dissociated to single-cell suspension by trypsinization, diluted in ES culture medium without mLIF and seeded in 25 μl hanging drops at a density of 100 or 400 cells per drop for analysis at Day 4 or Day 6 post-induction respectively.

(**b**) Representative brightfield images of embryoid bodies derived from *Prdm10*$^{F/F}$ and *Prdm10*$^{\Delta/\Delta}$ mESCs. *Prdm10*$^{\Delta/\Delta}$ cells form EBs that initially appear indistinguishable from controls (Day 2), but later on begin to disintegrate, showing morphological features of cell death (Day 4). Scale bar: 100 μm.

(c) Expression of lineage markers for all three germ layers in EBs harvested at indicated time-points, normalized to *Ubb* and presented as fold-change relative to undifferentiated mESCs (Day 0).

(d) Expression of ESC pluripotency markers in *Prdm10*$^{\Delta/\Delta}$ EBs, presented as mean ± s.d. fold-change relative to *Prdm10*$^{F/F}$ controls at indicated time-points.

Each point in (**c**–**d**) represents a biological replicate comprising approximately 30 pooled EBs, measured as the mean of two technical qPCR replicates. RNA was extracted using the Arcturus PicoPure RNA Isolation Kit (Applied Biosystems) and converted to cDNA using the High-Capacity cDNA Reverse Transcription Kit (Applied Biosystems).

Supplementary Figure 6

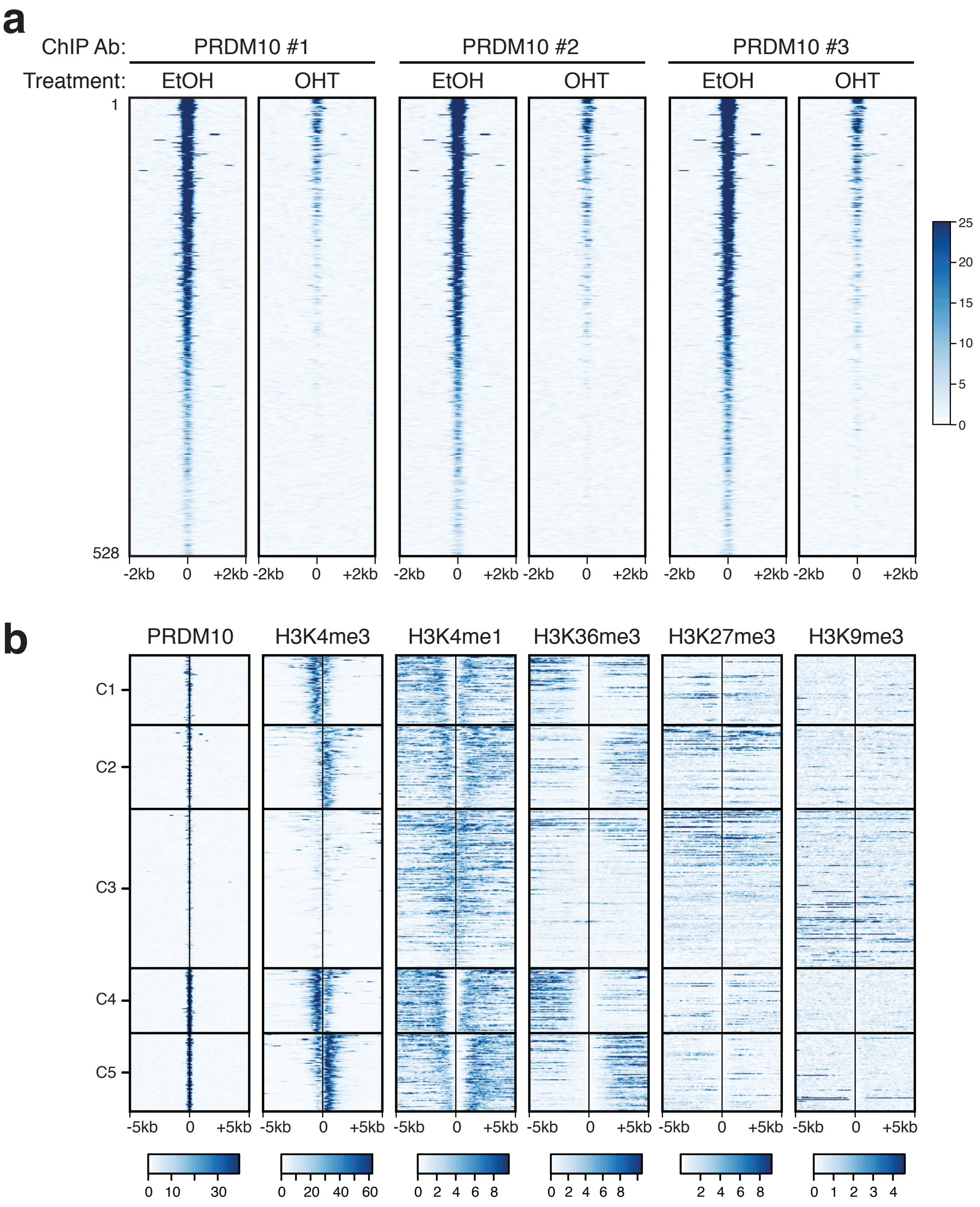

Supplementary Figure 6. ChIP-seq analysis of PRDM10 binding sites in mESCs.

(a) Heatmap representation of PRDM10 binding signal within ±2 kb of peak centers for control (EtOH) vs. PRDM10-depleted (OHT) mESCs. Data shown from three independent ChIP-seq datasets generated using different PRDM10 antibodies, denoted here as PRDM10 #1, #2 and #3.

(b) Clustering analysis of chromatin features associated with PRDM10-bound regions in mESCs reveals a majority of peaks overlapping actively transcribed gene promoters marked by H3K4me3 and H3K4me1, with H3K36me3 signals over adjacent gene bodies (clusters C1, C2, C4, C5). PRDM10-bound regions are largely depleted of repressive marks such as H3K27me3 and H3K9me3. Heatmaps were generated using publicly available mESC histone ChIP-seq data from ENCODE.

Supplementary Figure 7

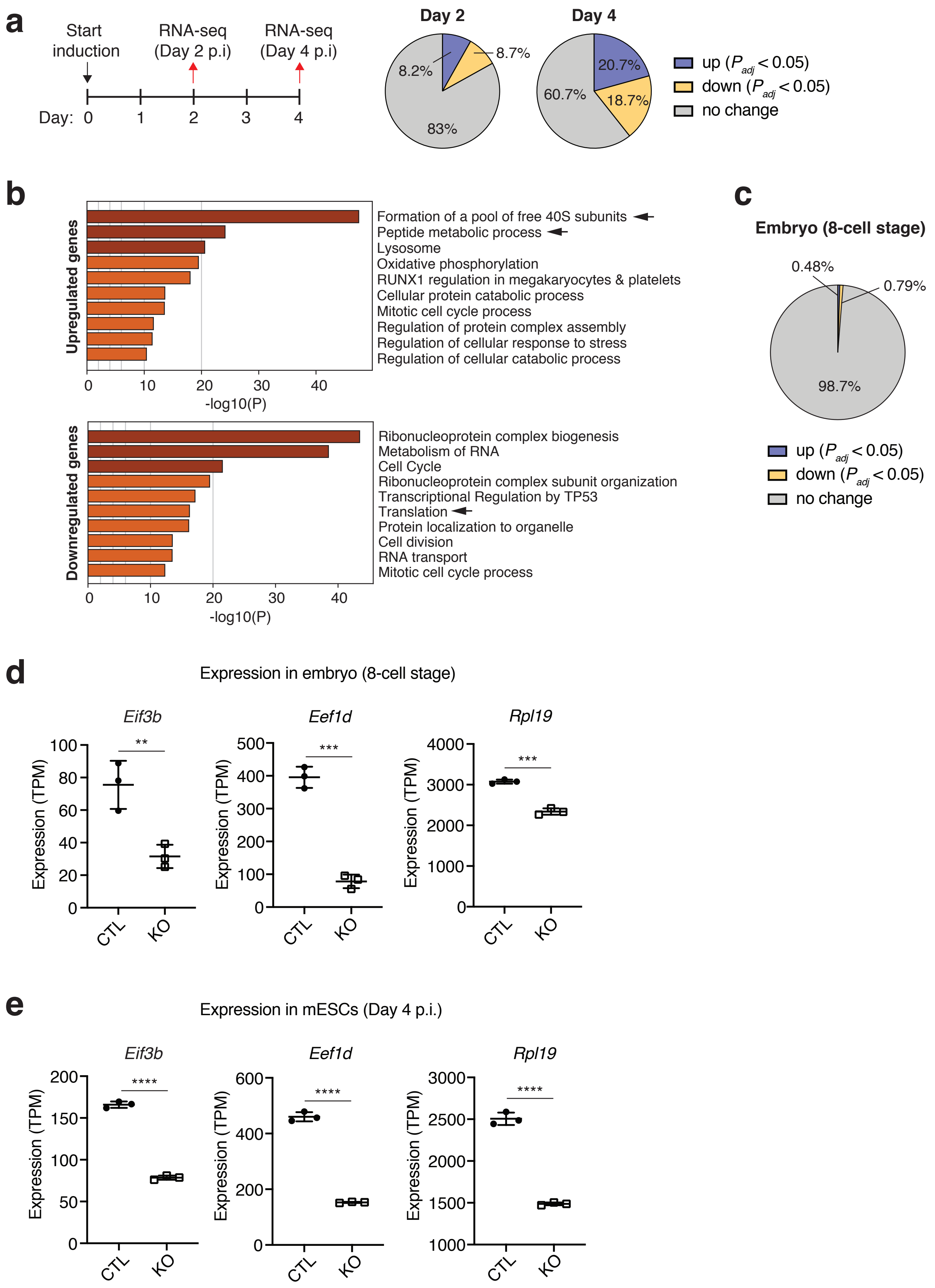

Supplementary Figure 7. Differential gene expression in *Prdm10*$^{\Delta/\Delta}$ mESCs and embryos compared to controls.

(a) Schematic outlining experimental workflow for RNA-seq analysis of *Prdm10*-deficient mESCs (*left*). Global differential gene expression in *Prdm10*$^{\Delta/\Delta}$ mESCs compared to *Prdm10*$^{F/F}$ controls ($P_{adj} < 0.05$), at Day 2 and Day 4 post-induction (*right*).

(b) GO analysis of all genes significantly up- or downregulated ($P_{adj} < 0.05$) in *Prdm10*$^{\Delta/\Delta}$ mESCs shows enrichment of terms related to ribosomal function and protein synthesis.

(c) Summary of differential gene expression ($P_{adj} < 0.05$) in *Prdm10*$^{\Delta/\Delta}$ vs. control 8-cell stage embryos.

(d) *Eif3b*, *Eef1d* and *Rpl19* transcript levels in 8-cell stage embryos from RNA-seq analysis, quantified as TPM (transcripts per million) values. Embryo genotypes were determined based on the presence (*Prdm10*$^{+/+}$ or *Prdm10*$^{\Delta/+}$ = CTL) or absence (*Prdm10*$^{\Delta/\Delta}$ = KO) of *Prdm10* exon 5 expression.

(e) *Eif3b*, *Eef1d* and *Rpl19* transcript levels in *Prdm10*$^{\Delta/\Delta}$ and control mESCs, analyzed by RNA-seq at Day 4 post-induction.

Supplementary Figure 8

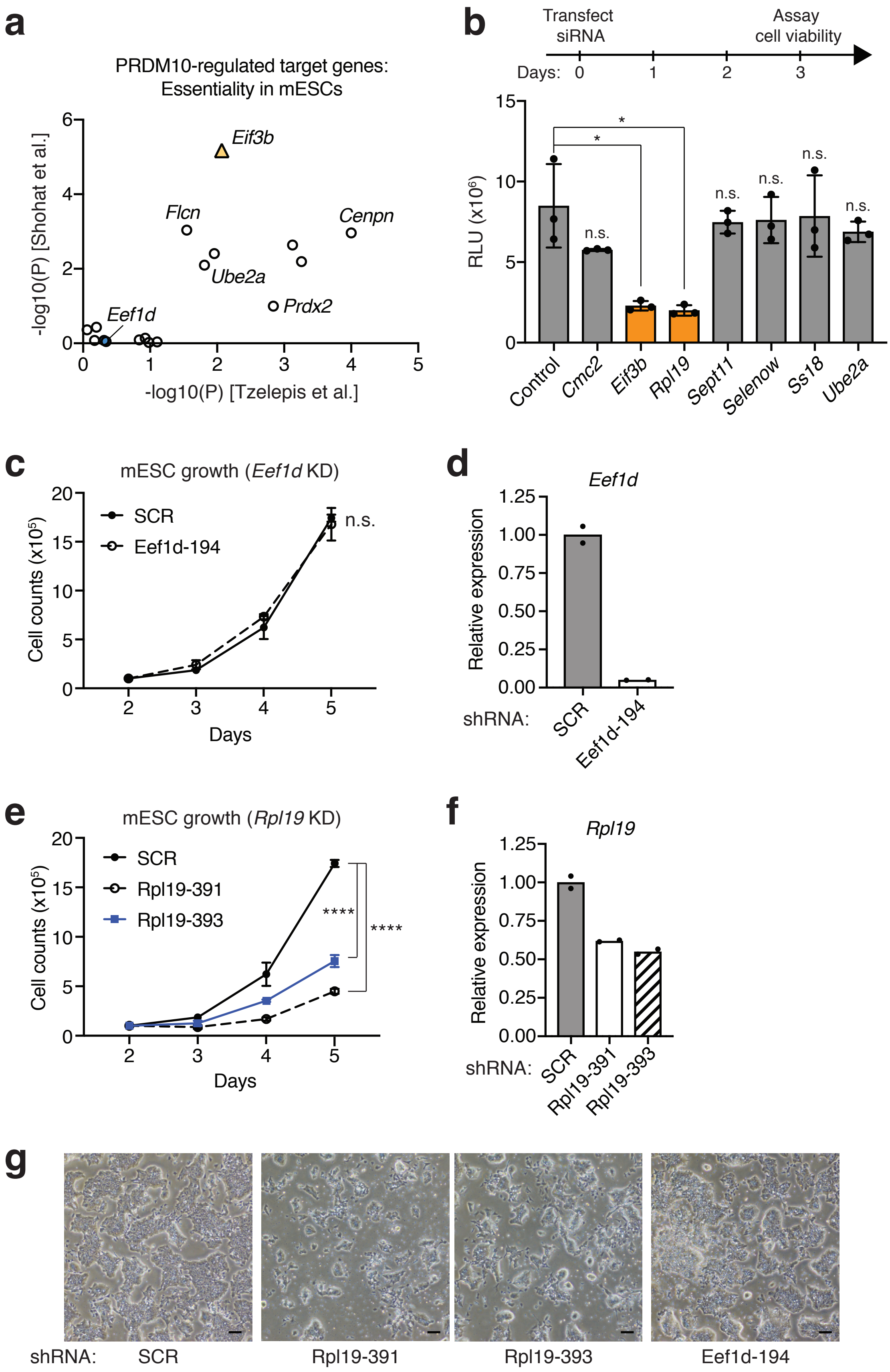

Supplementary Figure 8. Investigation of other PRDM10 target genes involved in translation.

(a) Analysis of gene essentiality in mESCs for the set of PRDM10 target genes defined in **Fig 3c–d**. For each gene, log-transformed negative selection P-value scores reported in two independent studies (Tzelepis et al.[1], Shohat et al.[2]) are plotted. *Eif3b* (yellow triangle) was scored highly as an essential gene in both screens, whereas *Eef1d* (blue circle) scored as non-essential.

(b) Candidate target genes were depleted in E14 mESCs by siRNA transfection, and viable cells measured 72 h post-transfection by CellTiterGlo assay. Data presented as mean ± s.d, $^*P < 0.05$, n.s., not significant; two-tailed unpaired Student's *t*-test. Each point represents 1 replicate sample, representative data shown from 1 out of 2 independent experiments.

(c) E14 mESCs were transduced with an shRNA construct targeting *Eef1d* (Eef1d-194), or non-targeting (SCR) control. After 2 days' culture under puromycin selection, cells were seeded in technical triplicate at constant density and cell counts measured at indicated time-points.

(d) qRT-PCR analysis showing efficient *Eef1d* knockdown in Eef1d-194 shRNA-transduced cells analyzed at day 5.

(e) Growth curves for E14 mESCs transduced with shRNA targeting *Rpl19* (Rpl19-391, Rpl19-393), vs. non-targeting (SCR) control.

(f) qRT-PCR analysis of *Rpl19* transcript knockdown in shRNA-transduced mESCs, analyzed at day 5.

(g) Representative brightfield images of shRNA-transduced cells, seeded at constant density at day 2 and analyzed at day 5.

Data in (**c**, **e**) presented as mean ± s.d, $n = 3$, ****$P < 0.0001$, n.s., not significant, two-way ANOVA with Tukey's multiple comparisons test. Expression in (**d**, **f**) presented relative to SCR controls, $n = 2$ biological replicates. Representative data from 1 of 2 independent experiments shown (**c**–**g**).

Supplementary Figure 9

a **b** **c**

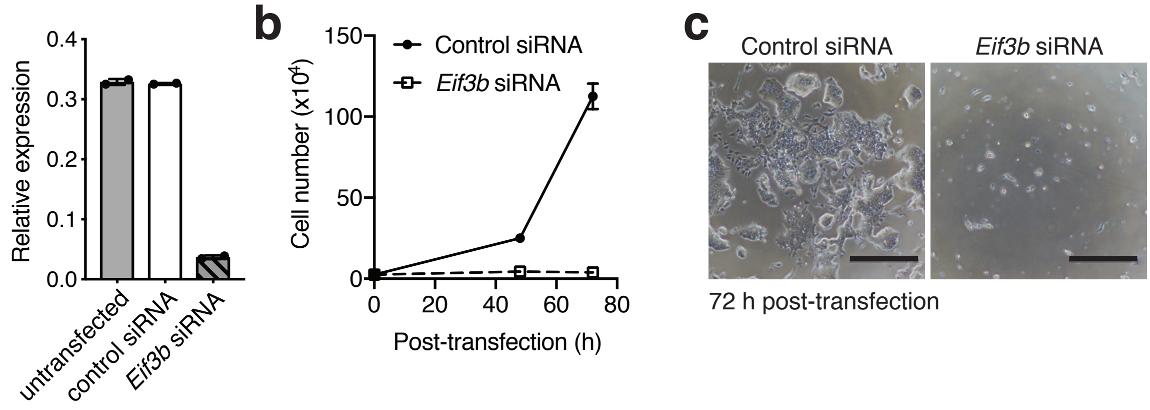

d

GCATTGTGCGGGCAGTCATTCTTGTGTGTGTTGGCGGCCCC**TGGTTGTCGGC**GAGCTCCTCGCG
TAATTTTGGAACCCTTCGTTCTTTTGCCCCGTCAGTGGACCGGTCTAAAATGCAAGACCTTACG
GGGGAGCGAAGCCAGTCATGTCAGTCAAGCAGTCTGTCTGATTAAAAGCAAAGCTTACAAAGAA
TCATGTGTGGAGCTTTCAGTTGTCTTGGCATTATTTTATGGTCACATATTTTGTCTTCATACAG
TTACGCGTGTTAATCATTTGAGAATCCACTTGATGCTCCCCAGATAGTACAGTCCTTCCCCGTA
GCGCCCGCTGGGAGAGGCAAAGGAACTTCTACCATCTTGGACGCAACTATTTCTTAGCTAGAAT
GGGGGAGCCACATATCCTTGTGAATTTATTTGCTACCTTAAAAAACCTTTTGGGCTATAAATAA
ACTTGCATATCTTGAAACTTATTATTTTAATTGTTTCTAGATTTTTATGCATATCTGAGTTTTC
TCGGGCTGATCGTGAGGAATGAGGGCGTGGGTGTCTCCCCCGAGCCACATCT**TGGTACGATCC**C
ATCGCTGCACGCACATGGCTCCGCGCTAGCGGCGCGCGGTGACTTCTGGGAGTCGGAAGCAGGG
CGGCCGAGCCGGGTGAGAAGCAGCGCGGGCCCATGCAGGACGCTGAGAACGTGGCGGTGCCCGA
GGCGGCCGAGGAGCGGGCTGAGCCCGCGAGGCAGCAGCCGGCCTCCGAGTCGCCGCCGACGGAC
GAGGCGGCAGGGTCTGGGGGGTCCGAAGTGGGGCAGACGGAAGACGCCGAAGA

Legend: P1 P2 Eif3b CDS

e

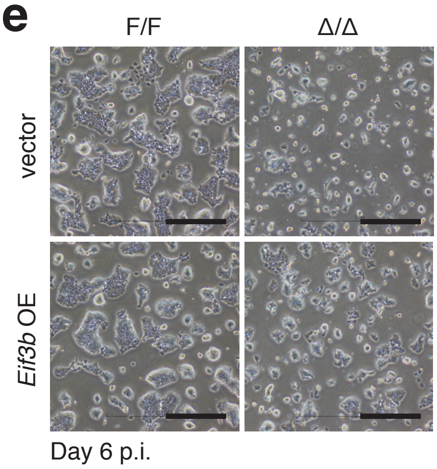

Supplementary Figure 9. *Eif3b* acts downstream of PRDM10 to mediate mESC survival.

(a) qRT-PCR analysis of *Eif3b* mRNA in E14 mESCs collected 48 h post-transfection with non-targeting control siRNA or *Eif3b*-specific siRNA. Expression normalized to *Ubb*. Each point represents a biological replicate; data pooled from two independent experiments.

(b) siRNA-mediated *Eif3b* knockdown results in strong inhibition of cell growth. E14 mESCs were transfected with siRNA, and cell numbers were measured at 48 h and 72 h post-transfection. *n* = 3 replicates.

(c) Representative brightfield images of E14 mESCs at 72 h after transfection with non-targeting control or *Eif3b* siRNA. Scale bar: 500 µm.

(d) *Eif3b* promoter sequence with P1 (red) and P2 (blue) subpeak regions highlighted. Closest consensus motif match in each subpeak is underlined. P1 and P2 were cloned separately into pGL4.23 for dual luciferase reporter assays.

(e) Representative images showing partial rescue of cell growth in *Prdm10*$^{\Delta/\Delta}$ mESCs expressing exogenous *Eif3b* compared to vector control, observed at Day 6 after *Prdm10* deletion. Scale bar: 500 µm.

Data shown as mean ± s.d. (**a** and **b**), representative of two (**a**–**c**) or three (**e**) independent experiments.

Supplementary Figure 10

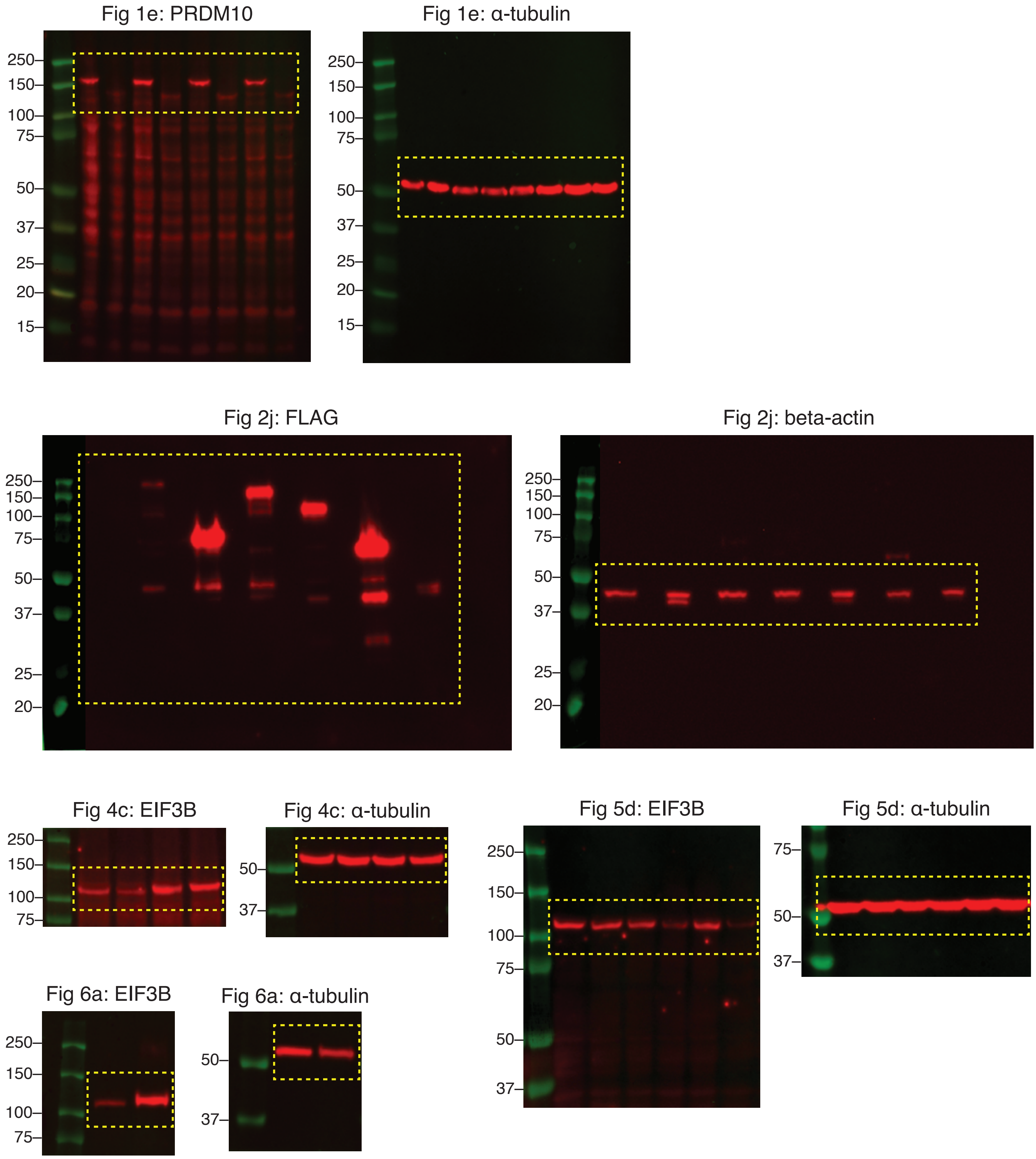

Supplementary Figure 10. Western blot images with molecular weight markers.

Yellow dashed boxes indicate area cropped for display in main figures.

Supplementary References

1. Tzelepis, K. *et al.* A CRISPR Dropout Screen Identifies Genetic Vulnerabilities and Therapeutic Targets in Acute Myeloid Leukemia. *Cell Reports* **17,** 1193–1205 (2016).
2. Shohat, S. & Shifman, S. Genes essential for embryonic stem cells are associated with neurodevelopmental disorders. *Genome Research* **29,** 1910–1918 (2019).
