# Supplementary material for: Global translation during early development depends on the essential transcription factor PRDM10
Source: Nat Commun. 2020 Jul 17;11:3603. doi: 10.1038/s41467-020-17304-3 (PMC7368010; doi:10.1038/s41467-020-17304-3)
Supplement: Supplementary file 3 — Reporting Summary [file 41467_2020_17304_MOESM3_ESM.pdf]

nature research

Corresponding author(s): Ernesto Guccione, Daniel Messerschmidt

Last updated by author(s): May 8, 2020

Reporting Summary

Nature Research wishes to improve the reproducibility of the work that we publish. This form provides structure for consistency and transparency in reporting. For further information on Nature Research policies, see our Editorial Policies and the Editorial Policy Checklist.

Statistics

For all statistical analyses, confirm that the following items are present in the figure legend, table legend, main text, or Methods section.

n/a	Confirmed	
☐	☒	The exact sample size (n) for each experimental group/condition, given as a discrete number and unit of measurement
☐	☒	A statement on whether measurements were taken from distinct samples or whether the same sample was measured repeatedly
☐	☒	The statistical test(s) used AND whether they are one- or two-sided<br>*Only common tests should be described solely by name; describe more complex techniques in the Methods section.*
☒	☐	A description of all covariates tested
☐	☒	A description of any assumptions or corrections, such as tests of normality and adjustment for multiple comparisons
☐	☒	A full description of the statistical parameters including central tendency (e.g. means) or other basic estimates (e.g. regression coefficient) AND variation (e.g. standard deviation) or associated estimates of uncertainty (e.g. confidence intervals)
☐	☒	For null hypothesis testing, the test statistic (e.g. F, t, r) with confidence intervals, effect sizes, degrees of freedom and P value noted<br>*Give P values as exact values whenever suitable.*
☒	☐	For Bayesian analysis, information on the choice of priors and Markov chain Monte Carlo settings
☒	☐	For hierarchical and complex designs, identification of the appropriate level for tests and full reporting of outcomes
☒	☐	Estimates of effect sizes (e.g. Cohen's d, Pearson's r), indicating how they were calculated

Our web collection on statistics for biologists contains articles on many of the points above.

Software and code

Policy information about availability of computer code

Data collection: The following open source software were used to analyze the data presented in this study.

Data analysis:
FastQC v0.11.5
bwa v0.7.13
Picard v2.10.6
samtools v1.2
SPP v1.13
IDR v2.0.4.2
ChIPpeakAnno v.3.16.1
Homer v4.10.4
deepTools v3.3.0
STAR v2.6.0c
RSEM v1.3.1
DESeq2 v1.22.2
R v3.5.1
SeqPlots v3.0.12
EnhancedVolcano v.1.0.1
IGV v2.4.14
Metascape
GraphPad Prism (v8)
FlowJo (v10)

For manuscripts utilizing custom algorithms or software that are central to the research but not yet described in published literature, software must be made available to editors and reviewers. We strongly encourage code deposition in a community repository (e.g. GitHub). See the Nature Research guidelines for submitting code & software for further information.

Data

Policy information about availability of data

All manuscripts must include a data availability statement. This statement should provide the following information, where applicable:

- Accession codes, unique identifiers, or web links for publicly available datasets
- A list of figures that have associated raw data
- A description of any restrictions on data availability

ChIP-seq and RNA-seq data sets generated in this study have been deposited in NCBI's Gene Expression Omnibus (GEO) under accession code GSE135022. All other data supporting the findings of this study are available from the corresponding authors upon reasonable request.

Field-specific reporting

Please select the one below that is the best fit for your research. If you are not sure, read the appropriate sections before making your selection.

☒ Life sciences ☐ Behavioural & social sciences ☐ Ecological, evolutionary & environmental sciences

For a reference copy of the document with all sections, see nature.com/documents/nr-reporting-summary-flat.pdf

Life sciences study design

All studies must disclose on these points even when the disclosure is negative.

Sample size	No statistical methods were used to predetermine the sample size.
Data exclusions	From our single embryo (8-cell stage) RNA-seq dataset, a single sample (RME807) was discarded due to poor library quality and extremely low read counts.
Replication	All experiments were performed and analyzed in three independent batches to ensure reproducibility. The findings reported in this study were successfully replicated.
Randomization	Mice used for timed matings were co-housed to minimize cage-to-cage variation and genotyped to ensure genetic identity. Female mice of varying ages between 6 to 12 weeks were used for timed matings, and the resulting embryos were pooled for analysis.
Blinding	For embryo experiments, the investigators were blinded to embryo identity during the initial process of visual evaluation and image acquisition as the genotyping process necessitated destruction of the embryo and could only be done after data collection. For the rest of this study, the investigators were not blinded to allocation during experiments and outcome assessment as most experiments required assessment of differences between mutant and control cell lines, which have to be identified and tracked by genotype.

Reporting for specific materials, systems and methods

We require information from authors about some types of materials, experimental systems and methods used in many studies. Here, indicate whether each material, system or method listed is relevant to your study. If you are not sure if a list item applies to your research, read the appropriate section before selecting a response.

Materials & experimental systems

n/a	Involved in the study	
☐	☒	Antibodies
☐	☒	Eukaryotic cell lines
☒	☐	Palaeontology and archaeology
☐	☒	Animals and other organisms
☒	☐	Human research participants
☒	☐	Clinical data
☒	☐	Dual use research of concern

Methods

n/a	Involved in the study	
☐	☒	ChIP-seq
☒	☐	Flow cytometry
☒	☐	MRI-based neuroimaging

Antibodies

Antibodies used	1. anti-PRDM10 rabbit polyclonal; Bethyl Laboratories, catalog # A303-204A, lot # A303-204-1<br>2. anti-EIF3B rabbit polyclonal; Bio-Rad, catalog # VPA00380, lot # 160411<br>3. anti-alpha-tubulin mouse monoclonal, clone B-5-1-2; Sigma, catalog # T5168, lot # 103M4773V<br>4. anti-beta-actin mouse monoclonal, clone C4; Santa Cruz, catalog # sc-47778, lot # J1512<br>5. anti-CDX2 rabbit polyclonal; Abcam, catalog # ab88129, lot # GR297577-1<br>6. anti-NANOG rabbit polyclonal; ReproCELL, catalog # RCAB002P-F, lot # C01PL06

7. anti-OCT3/4 mouse monoclonal; Santa Cruz, catalog # sc-5279
8. anti-FLAG M2 mouse monoclonal; Sigma, catalog # F1804

Validation

1. Validated for ChIP and Western blot. Tested for reactivity against human and mouse and validated by IP/WB (supplier's website). Passed ENCODE consortium primary characterization QC check (antibody ID: ENCAB587QKH) and used in PRDM10 ChIP-seq on human K562 cells (ENCODE dataset ID: ENCSR120MPG). Antibody specificity was further confirmed in-house by western blot and ChIP with PRDM10 knockout cells as negative control.
2. Reactive against human and mouse EIF3B; validated for WB (supplier's website).
3. Reactive against multiple species including mouse; recognizes an epitope located at the C-terminal end of the α-tubulin isoform in a variety of organisms, validated for WB and extensively cited in multiple publications (supplier's website).
4. Reactive against beta-actin from multiple species including mouse; may cross-react with other actin isoforms in higher vertebrates; validated for multiple applications including WB (supplier's website).
5. Reactive against human and mouse; validated for IHC and WB (supplier's website).
6. Reactive against mouse Nanog; validated for IF and WB (supplier's website).
7. Reactive against mouse and human OCT3/4; validated for multiple applications including IF and extensively cited in multiple publications (supplier's website).
8. Highly sensitive and specific detetion of FLAG peptide sequence validated by WB, IP and IF (supplier's website).

Eukaryotic cell lines

Policy information about cell lines

Cell line source(s)

1) Mouse embryonic stem cell lines (Prdm10F/F, Prdm10F/+; Rosa26-CreERT2), derived from E3.5 mouse blastocysts as described in this study
2) Human embryonic kidney cell line (HEK293T), from ATCC

Authentication

1) Molecular validation of mESC lines was performed as described in this study
2) HEK293T cells were derived from ATCC; no further authentication was performed by the authors

Mycoplasma contamination

All cell lines used in this study tested negative for mycoplasma contamination by a PCR-based assay.

Commonly misidentified lines
(See ICLAC register)

No commonly misidentified cell lines were used.

Animals and other organisms

Policy information about studies involving animals; ARRIVE guidelines recommended for reporting animal research

Laboratory animals

Species: Mus musculus
Strain background: C57BL/6
Source: Prdm10tm1a(EUCOMM)Hmgu targeted ES clone
Line used: Prdm10 lacZ/+, Prdm10 del/+ (both Prdm10 +/-)
Gender used in experiments: Males and females (Prdm10 +/-) were used in intercrosses

Wild animals

This study did not involve wild animals.

Field-collected samples

This study did not involve samples collected in the field.

Ethics oversight

All animal work was performed in compliance with the Institutional Animal Care and Use Committee (IACUC) protocols #151042 and #181393, with the approval of the Biological Resource Centre (BRC), A*STAR.

Note that full information on the approval of the study protocol must also be provided in the manuscript.

ChIP-seq

Data deposition

☒ Confirm that both raw and final processed data have been deposited in a public database such as GEO.

☒ Confirm that you have deposited or provided access to graph files (e.g. BED files) for the called peaks.

Data access links
May remain private before publication.

Series GSE135020
https://www.ncbi.nlm.nih.gov/geo/query/acc.cgi?acc=GSE135020

Files in database submission

Raw data:
merge_fastqs_R1_CHE228_NS001-SR-R00366_L001_R1.merged.fastq
merge_fastqs_R1_CHE229_NS001-SR-R00366_L001_R1.merged.fastq
merge_fastqs_R1_CHE230_NS001-SR-R00366_L001_R1.merged.fastq
merge_fastqs_R1_CHE231_NS001-SR-R00366_L001_R1.merged.fastq
merge_fastqs_R1_CHE232_NS001-SR-R00366_L001_R1.merged.fastq
merge_fastqs_R1_CHE233_NS001-SR-R00366_L001_R1.merged.fastq
merge_fastqs_R1_CHE234_NS001-SR-R00366_L001_R1.merged.fastq
merge_fastqs_R1_CHE235_NS001-SR-R00366_L001_R1.merged.fastq

Processed data:
CHE228_NS001-SR-R00366_L001_R1.merged.nodup_x_ctl_for_rep1.fc.signal.bigwig
CHE229_NS001-SR-R00366_L001_R1.merged.nodup_x_ctl_for_rep2.fc.signal.bigwig
CHE230_NS001-SR-R00366_L001_R1.merged.nodup_x_ctl_for_rep3.fc.signal.bigwig
CHE232_NS001-SR-R00366_L001_R1.merged.nodup_x_ctl_for_rep1.fc.signal.bigwig
CHE233_NS001-SR-R00366_L001_R1.merged.nodup_x_ctl_for_rep2.fc.signal.bigwig
CHE234_NS001-SR-R00366_L001_R1.merged.nodup_x_ctl_for_rep3.fc.signal.bigwig
peaks.KO_IDR.optimal.regionPeak
peaks.WT_IDR.optimal.regionPeak

Genome browser session (e.g. UCSC)

No longer applicable (final submission).

Methodology

Replicates

3 independent ChIP-seq replicates were performed, each with a different polyclonal anti-PRDM10 antibody as described in the study.

Sequencing depth

All samples were sequenced as single-end 75bp reads to generate >20M raw reads per library.

Library ID/ Sample/ total reads/ mapped reads
CHE228/ WT_Prdm10 ChIP 1/ 22273075/ 21351941
CHE229/ WT_Prdm10 ChIP 2/ 20817202/ 19971948
CHE230/ WT_Prdm10 ChIP 3/ 21946096/ 21041719
CHE231/ WT_Input/ 20328972/ 19536086
CHE232/ KO_Prdm10 ChIP 1/ 22381042/ 21524772
CHE233/ KO_Prdm10 ChIP 2/ 21133292/ 20298482
CHE234/ KO_Prdm10 ChIP 3/ 21824519/ 20969707
CHE235/ KO_Input/ 21283916/ 20456165

Antibodies

1. anti-PRDM10 rabbit polyclonal; Bethyl Laboratories, catalog # A303-204A, lot # A303-204-1
2. anti-PRDM10 rabbit polyclonal; raised against PRDM10 C-terminal peptide (SEIQMMTLPPGQFVITDSGC), generated in-house from 2 separate immunizations

Peak calling parameters

We used the SPP peak caller in transcription factor mode according to the ENCODE ChIP-seq pipeline v1.1.7 (https://github.com/ENCODE-DCC/chip-seq-pipeline2).

Data quality

Sequencing quality was assessed using FastQC. Other ChIP-seq quality metrics were evaluated as part of the ENCODE ChIP-seq pipeline v1.1.7 (https://github.com/ENCODE-DCC/chip-seq-pipeline2); all libraries passed library complexity QC (NRF>0.9, PBC1>0.9, PBC2>10) and peak reproducibility QC (self-consistency ratio<2, rescue ratio<2).

Software

bwa v0.7.13
Picard v2.10.6
samtools v1.2
SPP v1.13
IDR v2.0.4.2
ChIPpeakAnno v.3.16.1
