# Supplementary material for: Global translation during early development depends on the essential transcription factor PRDM10
Source: Nat Commun. 2020 Jul 17;11:3603. doi: 10.1038/s41467-020-17304-3 (PMC7368010; doi:10.1038/s41467-020-17304-3)
Supplement: Supplementary file 4 — Description of Additional Supplementary Files [file 41467_2020_17304_MOESM4_ESM.pdf]

Description of Additional Supplementary Files

File Name: Supplementary Data 1
Description: PRDM10 ChIP-seq peaks

File Name: Supplementary Data 2
Description: Differential gene expression for KO vs. WT mESCs at Day 2 post-deletion

File Name: Supplementary Data 3
Description: Differential gene expression for KO vs. WT mESCs at Day 4 post-deletion

File Name: Supplementary Data 4
Description: GO enrichment analysis of genes differentially expressed (KO vs. WT, padj<0.05) at Day 4

File name: Supplementary Data 5
Description: Gene expression in 8-cell stage embryos (MUT vs. CTL)

File name: Supplementary Data 6
Description: Genes associated with PRDM10 binding sites

File name: Supplementary Data 7
Description: Genes bound by PRDM10 and differentially expressed in Prdm10-null mESCs/ embryos (padj<0.05, fold-change>2)

File name: Supplementary Data 8
Description: Oligonucleotide sequences
